# Deuterium Oxide (D_2_O) Induces Early Stress Response Gene Expression and Impairs Growth and Metastasis of Experimental Malignant Melanoma

**DOI:** 10.3390/cancers13040605

**Published:** 2021-02-03

**Authors:** Jana Jandova, Anh B. Hua, Jocelyn Fimbres, Georg T. Wondrak

**Affiliations:** Department of Pharmacology and Toxicology, College of Pharmacy & UA Cancer Center, University of Arizona, Tucson, AZ 85724, USA; jjandova@arizona.edu (J.J.); anhhua@arizona.edu (A.B.H.); jocelynf@arizona.edu (J.F.)

**Keywords:** malignant melanoma, deuterium oxide, heavy water, SCID mouse metastasis model, A375 melanoma xenograft model, cellular stress response, A375-luciferase reporter cells, transwell invasion

## Abstract

**Simple Summary:**

Systemic administration of deuterium oxide (‘heavy water’) has shown promise in suppressing tumor growth and metastasis in mammalian cancer models, but no detailed molecular studies have revealed specific molecular pathways mediating cancer-cell-directed activities. Here, for the first time, transcriptomic analysis complemented by in vivo efficacy experiments have addressed this unresolved topic.

**Abstract:**

There are two stable isotopes of hydrogen, protium (^1^H) and deuterium (^2^H; D). Cellular stress response dysregulation in cancer represents both a major pathological driving force and a promising therapeutic target, but the molecular consequences and potential therapeutic impact of deuterium (^2^H)-stress on cancer cells remain largely unexplored. We have examined the anti-proliferative and apoptogenic effects of deuterium oxide (D_2_O; ‘heavy water’) together with stress response gene expression profiling in panels of malignant melanoma (A375^V600E^, A375^NRAS^, G361, LOX-IMVI), and pancreatic ductal adenocarcinoma (PANC-1, Capan-2, or MIA PaCa-2) cells with inclusion of human diploid Hs27 skin fibroblasts. Moreover, we have examined the efficacy of D_2_O-based pharmacological intervention in murine models of human melanoma tumor growth and metastasis. D_2_O-induction of apoptosis was substantiated by AV-PI flow cytometry, immunodetection of PARP-1, and pro-caspase 3 cleavage, and rescue by pan-caspase inhibition. Differential array analysis revealed early modulation of stress response gene expression in both A375 melanoma and PANC-1 adenocarcinoma cells elicited by D_2_O (90%; ≤6 h) (upregulated: *CDKN1A*, *DDIT3*, *EGR1*, *GADD45A*, *HMOX1*, *NFKBIA*, or *SOD2* (up to 9-fold; *p* < 0.01)) confirmed by independent RT-qPCR analysis. Immunoblot analysis revealed rapid onset of D_2_O-induced stress response phospho-protein activation (p-ERK, p-JNK, p-eIF2α, or p-H2AX) or attenuation (p-AKT). Feasibility of D_2_O-based chemotherapeutic intervention (drinking water (30% *w*/*w*)) was demonstrated in a severe combined immunodeficiency (SCID) mouse melanoma metastasis model using luciferase-expressing A375-Luc2 cells. Lung tumor burden (visualized by bioluminescence imaging) was attenuated by D_2_O, and inhibition of invasiveness was also confirmed in an in vitro Matrigel transwell invasion assay. D_2_O supplementation also suppressed tumor growth in a murine xenograft model of human melanoma, and median survival was significantly increased without causing adverse effects. These data demonstrate for the first time that systemic D_2_O administration impairs growth and metastasis of malignant melanoma through the pharmacological induction of deuterium (^2^H)-stress.

## 1. Introduction

There are two stable isotopes of hydrogen, protium (^1^H) and deuterium (^2^H (D)). Since its initial discovery as a natural heavy isotope variant of dihydrogen oxide (^1^H_2_O), extensive research has focused on the toxicological, biochemical, and pharmacological effects of deuterated water (^2^H_2_O (D_2_O, also referred to as ‘heavy water’)) [1]. The pharmacokinetics of tissue deuteration through systemic administration of D_2_O have been studied in much detail in mammalian systems, including mice, and feasibility and toxicological consequences of long-term systemic administration of D_2_O through drinking water supplementation (20% and above) have been investigated [2,3,4,5,6,7,8,9,10]. Administration of D_2_O reaching up to 23% has been documented in investigational human studies for numerous purposes including determination of body water composition and use of D_2_O as a potential modulator for neutron capture therapy (in the context of nuclear medicine) [8,10,11,12]. Biological effects of D_2_O (including anti-mitotic and apoptogenic activities) are generally attributed to altered isotopic and solvent properties, associated with an increased strength of deuterium-based hydrogen bonds impacting (i) conformational stability of proteins and (ii) fidelity of nucleic acid base pairing (relevant to replication, transcription, and translation processes) [10,13,14,15,16,17]. Specific molecular targets potentially mediating pharmacological effects of D_2_O such as modulation of water channels (aquaporin 11 (AQP11)), inhibition of L-type (dihydropyridine-sensitive) calcium channels, modulation of cytoskeletal components (microtubules and actomyosin), and modulation of cellular signaling have been explored [10,16,17,18,19,20,21,22,23,24,25,26].

Importantly, shortly after its initial discovery by Urey in 1932, cancer-directed effects of D_2_O (administered systemically) have been examined in vivo, and inhibitory effects on murine tumor growth were described as early as 1938, documenting growth inhibition of implanted mammary carcinoma and lymphosarcoma using D_2_O drinking water supplementation (20–40% (*v*/*v*), over a period of nineteen days) [1,2]. Cumulative evidence now confirms tumor-directed activity of D_2_O supplementation in murine cancer models including pancreatic, colorectal, and squamous cell carcinoma [2,3,5,6,7,9]. However, the specific mechanisms underlying D_2_O-associated chemotherapeutic effects targeting cancer cells remain largely unexplored. Indeed, only limited information exists on D_2_O-induced early cellular stress responses assessed at the mRNA and protein levels, even though numerous studies have substantiated anti-proliferative, anti-mitotic, and apoptogenic effects of D_2_O on cultured cancer cells [10,27,28,29,30,31,32].

Cellular stress response dysregulation in cancer represents both a major pathological driving force and a promising therapeutic target [10,33,34]. Here, using a panel of cultured melanoma and pancreatic ductal adenocarcinoma cells we have profiled D_2_O-induced apoptogenicity and early stress response examined by gene expression array analysis and phosphoprotein immunodetection. We also document, for the first time, the chemotherapeutic effects of systemic D_2_O administration targeting malignant melanoma employing murine models of metastasis and tumor growth. 

## 2. Results

### 2.1. D_2_O Exposure Induces Apoptotic Cell Death in a Panel of Cultured Human Malignant Melanoma Cells

First, impairment of cellular viability in response to D_2_O exposure was monitored using flow cytometric analysis of annexin V-PI-stained cultured melanoma cells. To this end, cells were cultured in identical media that differed only by H/D isotope composition, prepared by reconstituting powdered medium using either pure H_2_O or H_2_O/D_2_O mixtures (containing increasing proportions of D_2_O: 0, 4.5, 9, 27, 45, and 90% complete water content).

**Figure 1:**
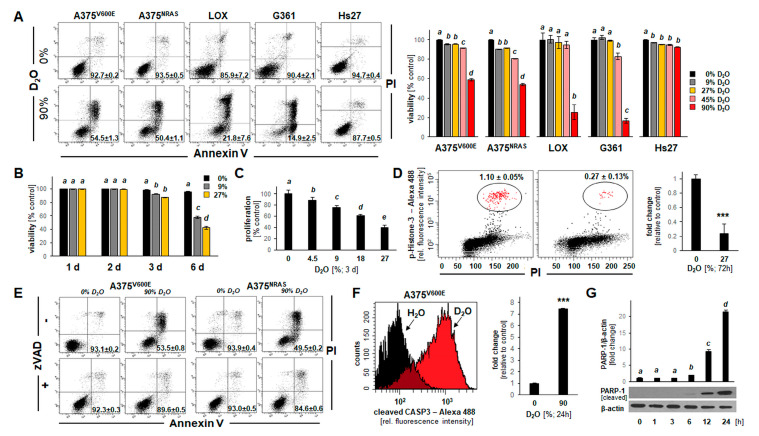
D_2_O-induced apoptosis in a panel of human malignant melanoma cells (A375^V600E^, A375^NRAS^, LOX-IMVI, and G361). (**A**) Impairment of cellular viability in response to culture in D_2_O (90%, 24 h) was monitored using flow cytometric analysis (annexin V-PI staining). Numbers in quadrants indicate percentage of viable cells (AV-negative, PI-negative) from a total of gated cells (mean ± SD, *n* = 3). Bar graph (right panel) indicates dose response of impaired cell viability (D_2_O (≤90%, 24 h)) (*n* = 3). Human Hs27 dermal fibroblasts exposed to D_2_O served as non-transformed, non-malignant controls. (**B**) Impairment of cellular viability in response to long term exposure to D_2_O (27%, ≤6 days). Bar graph depicts dose response and time course ((≤6 days); (*n* = 3)). (**C**) Impairment of cellular proliferation (A375) in response to culture in D_2_O (≤27%, 3 days). (**D**) M-phase depletion as a function of culture in D_2_O (27%, 3 days) as assessed by phospho-histone H3 flow cytometry: individual histograms representative of three repeats (left panel); right panel: bar graph depiction of numerical analysis (*n* = 3; *p* *** < 0.001). (**E**) D_2_O-induced (90%, 24 h) cell death (A375^V600E^ versus A375^NRAS^) in the absence or presence of zVAD-fmk (40 µM). (**F**) D_2_O-induced (90%, 24 h) induction of pro-caspase 3 cleavage as examined in A375^V600E^ cells by flow cytometry (left panel: representative histograms; right panel: bar graph depiction of numerical analysis (*n* = 3; *p* *** < 0.001). (**G**) Time course of PARP-1 cleavage in response to D_2_O-exposure (90%, ≤24 h) in A375 cells (bottom panel: immunoblot; top panel: bar graph depiction of numerical analysis (*n* = 3).

In a panel of cultured human malignant melanoma cells (A375^V600E^, A375^NRAS^, LOX-IMVI, or G361), it was observed that culture in D_2_O (90%; 24 h) induces apoptosis (Figure 1A). A more detailed dose-response analysis (D_2_O (≤90%, 24 h)) indicated that at D_2_O concentrations lower than 45%, viability was maintained over the duration of the observation period (Figure 1A, bar graph; means without a common letter differ (*p* < 0.05); see statistical analysis (Section 4.15) for details). Remarkably, human diploid Hs27 fibroblasts (serving as a non-transformed, non-malignant control) remained viable even if exposed to the highest D_2_O concentration (90%, 24 h).

Next, the effects of long-term exposure to D_2_O (9–27%, ≤6 d) on cellular viability were examined indicating that melanoma cell death can be induced at low D_2_O concentrations that were devoid of cytotoxicity observed over a 24 h period (Figure 1B). Likewise, inhibition of A375 cell proliferation occurred at D_2_O concentrations as low as 4.5% (Figure 1C), and significant M-phase depletion by more than 75% (examined by (Ser10) phospho-histone H3 flow cytometry (Figure 1D)) was observable in response to D_2_O exposure.

Next, in order to substantiate an apoptotic mode of cell death that might underlie D_2_O-induced (90%, 24 h) cytotoxicity, experiments were performed in the absence or presence of the pan-caspase inhibitor zVAD-fmk (40 µM) (Figure 1E). Strikingly, pan-caspase inhibition rescued A375 cells from D_2_O-induced (90%, 24 h) impairment of viability. Likewise, examination of D_2_O-induced (90%, 24 h) induction of pro-caspase 3 cleavage was conducted by flow cytometry (Figure 1F), confirming D_2_O induction of apoptosis. Finally, the time course of PARP-1 cleavage in response to D_2_O exposure (90%, ≤24 h) was examined in A375 cells (bottom panel: immunoblot analysis; top panel: bar graph depiction of numerical analysis (Figure 1G and Figure S1)). Taken together, these data indicate that D_2_O can induce cell death that occurs by induction of apoptosis. 

### 2.2. D_2_O Exposure Induces Apoptotic Cell Death in a Panel of Human Pancreatic Ductal Adenocarcinoma Cells (PANC-1, MIA PaCa-2, and Capan-2)

In order to explore the potential chemotherapeutic utility of D_2_O exposure on non-melanoma cancer cells, D_2_O-induced impairment of cellular viability was examined using a panel of pancreatic ductal adenocarcinoma (PDAC; PANC-1, MIA PaCa-2, and Capan-2).

**Figure 2:**
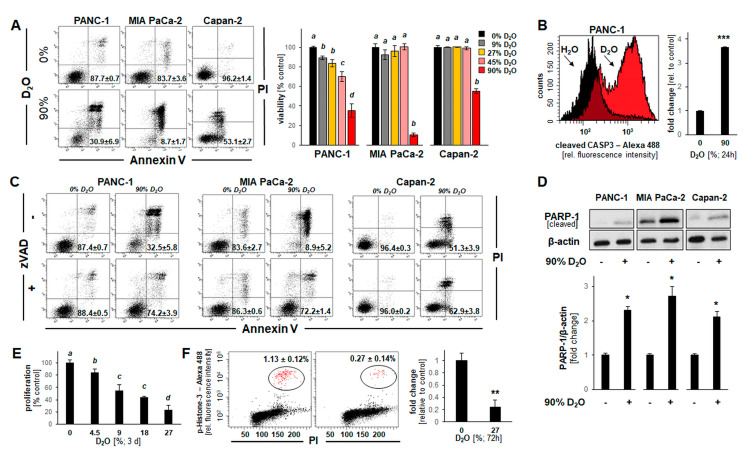
D_2_O-induced apoptosis in a panel of human pancreatic ductal adenocarcinoma cells (PANC-1, MIA PaCa-2, and Capan-2). (**A**) Loss of cellular viability in response to D_2_O (90%, 24 h) as analyzed in Figure 1A. (**B**) D_2_O-induced (90%, 24 h) induction of pro-caspase 3 cleavage examined in PANC-1 cells by flow cytometry (left panel: representative histograms; right panel: bar graph depiction of numerical analysis (*n* = 3; p *** < 0.001). (**C**) D_2_O-induced (90%, 24 h) cell death examined in the absence or presence of zVAD-fmk (40 µM). (**D**) PARP-1 cleavage in response to D_2_O-exposure (90%, 24 h) observable in pancreatic ductal adenocarcinoma (PDAC) cells by immunoblot analysis; bar graph depiction summarizes densitometric analysis (*n* = 3; p* < 0.05). (**E**) Impairment of cellular proliferation (PANC-1) by culture in D_2_O (≤27%, 3 days). (**F**) M-phase depletion as a function of culture in D_2_O (27%, 3 days) as assessed and analyzed in Figure 1D (*n* = 3; p ** < 0.01).

First, impairment of viability was monitored using flow cytometric analysis of annexin V-PI-stained PDAC cells. After observing that culture in D_2_O (90%, 24 h) induces apoptosis (Figure 2A), a detailed dose-response analysis (D_2_O (≤90%, 24 h)) indicated that at D_2_O concentrations lower than 45%, viability was maintained over the duration of the observation period (Figure 1A, bar graph). Likewise, examination of D_2_O-induced (90%, 24 h) cell death revealed pronounced induction of pro-caspase 3 cleavage (Figure 2B). As observed with cultured melanoma cells, an apoptotic mode of cell death underlying D_2_O-induced (90%, 24 h) impairment of cell viability was further supported by rescue using zVAD-fmk (40 µM) (Figure 2C). Likewise, PARP-1 cleavage was detected in PDAC cells in response to D_2_O exposure (90%, 24 h) (Figure 2D and Figure S2). Finally, as observed in melanoma cell lines (Figure 1C,D), D_2_O concentrations as low as 4.5% (72 h exposure time) were associated with significant inhibition of proliferation (Figure 2E). Moreover, pronounced M-phase depletion was observable upon prolonged exposure (27% D_2_O, 72 h; Figure 2F).

Taken together, these data indicate that D_2_O exposure can inhibit proliferation and induce apoptosis in a panel of cultured PDAC cells as characterized by AV/PI-positivity, zVADfmk-rescue, and proteolytic cleavage of pro-caspase 3 and PARP-1.

### 2.3. Array Analysis Reveals D_2_O-Induced Early Stress Response Gene Expression Changes Observable in Both Cultured Malignant Melanoma (A375) and Pancreatic Ductal Adenocarcinoma (PANC-1) Cells

**Figure 3:**
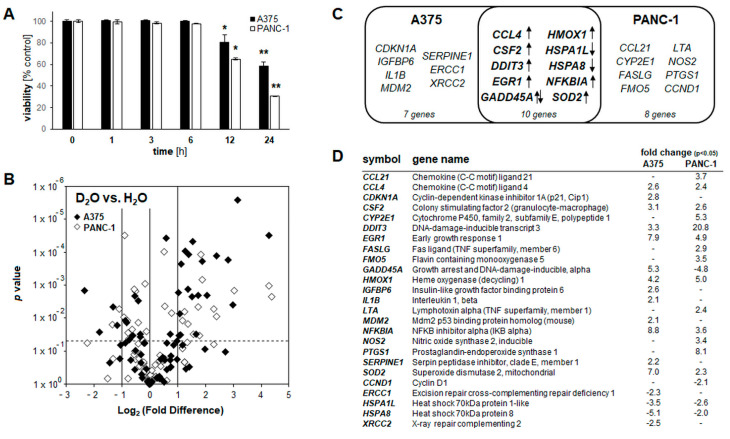
D_2_O-induced early stress response gene expression comparing melanoma (A375) and pancreatic ductal adenocarcinoma (PANC-1) cells. (**A**) Time-course analysis of cell viability impairment assessed by flow cytometry (performed as in Figure 1A) in A375 and PANC-1 cells cultured in 90% D_2_O (≤24 h; *n* = 3; p * < 0.05; p ** < 0.01). (**B**) Volcano plot depicting differential gene expression (untreated versus D_2_O-exposed (90%, 6 h)) as identified by the Human Stress and Toxicity PathwayFinder^TM^ PCR Array technology (cut off criteria: expression differential >2; *p*-value ≤ 0.05; *n* = 3; A375 (black diamond); PANC-1 (empty diamond). (**C**) Comparative gene expression array analysis in Venn diagram depiction; in the overlapping region, single arrow indicates congruent up- or downregulation, and double arrows indicate opposing expression changes between cell lines. (**D**) Comparative gene expression array analysis with total number of genes per group as summarized numerically (A375 vs. PANC-1; D_2_O exposed as in panel B).

In an attempt to further explore cellular responses elicited by D_2_O we performed differential gene expression analysis, examining and comparing the effects of D_2_O exposure on malignant melanoma (A375) and pancreatic ductal adenocarcinoma (PANC-1) cells (Figure 3). Time-course analysis indicated that cells exposed to a lethal concentration of D_2_O (90%) displayed a pronounced loss of viability upon prolonged exposure (24 h) while maintaining full viability at an early time point (6 h), an observation made in both cell lines (Figure 3A).

This specific dose regimen (90% D_2_O; 6 h continuous exposure) was therefore chosen to profile and compare D_2_O-induced cellular effects interrogating expression of 84 genes (Human Stress and Toxicity PathwayFinder^TM^ RT^2^ Profiler^TM^ PCR Array). In A375 melanoma cells, significant expression changes were detected at the mRNA level affecting nineteen genes (upregulated: *CCL4*, *CDKN1A*, *CSF2*, *DDIT3*, *DNAJB4*, *EGR1*, *GADD45A*, *HMOX1*, *HSPE1*, *IGFBP6*, *IL1B*, *MDM2*, *NFKBIA*, *SERPINE1*, *SOD2*; downregulated: *ERCC1*, *HSPA1L*, *HSPA8*, *XRCC2*; up to 9-fold; *p* < 0.05; Figure 3B: volcano plot; Figure 3D: table)). Likewise, in PANC-1 pancreatic ductal adenocarcinoma cells, gene expression changes were detected at the mRNA level affecting eighteen genes (upregulated: *CCL21*, *CCL4*, *CRYAB*, *CSF2*, *CYP2E1*, *DDIT3*, *EGR1*, *FASLG*, *FMO5*, *HMOX1*, *LTA*, *NFKBIA*, *NOS2*, *PTGS1*, *SOD2*; downregulated: *CCND1*, *GADD45A*, *HSPA1L*, *HSPA8* (up to 21-fold; *p* < 0.05; Figure 3B: volcano plot; Figure 3D: table)).

Strikingly, in response to exposure to lethal D_2_O concentrations a limited number of gene expression changes (detectable at an early time point (6 h) at which cells displayed full viability) were shared between A375 and PANC-1 cells (as indicated by comparative Venn diagram analysis (Figure 3C)). The following ten D_2_O-induced stress response gene expression changes were observed in both A375 and PANC-1 cells (fold change): oxidative stress ((*HMOX1* encoding heme oxygenase-1 (A375: 4.2; PANC-1: 5.0) and *SOD2* encoding mitochondrial superoxide dismutase 2 (A375: 7.0; PANC-1: 2.3)), genotoxic stress (*GADD45A* encoding growth arrest and DNA damage-inducible, alpha (A375: 5.3; PANC-1: −4.8)), endoplasmic reticulum stress (*DDIT3* encoding CHOP (C/EBP homologous protein 3)(A375: 3.3; PANC-1: 20.8)), pro-apoptotic tumor suppression (*EGR1* encoding early growth response 1 (A375: 7.9; PANC-1: 4.9)), modulation of NFκB inflammatory signaling (*NFKBIA* encoding IkBα (A375: 8.8; PANC-1: 3.6)), and cellular heat shock response (*HSPA1L* encoding Hsp70 protein 1-like (A375: -3.5; PANC-1: −2.6) and; HSPA8 encoding Hsp70 protein 8: (A375: −5.1; PANC-1: −2.0)) (Figure 3C,D).

**Figure 4:**
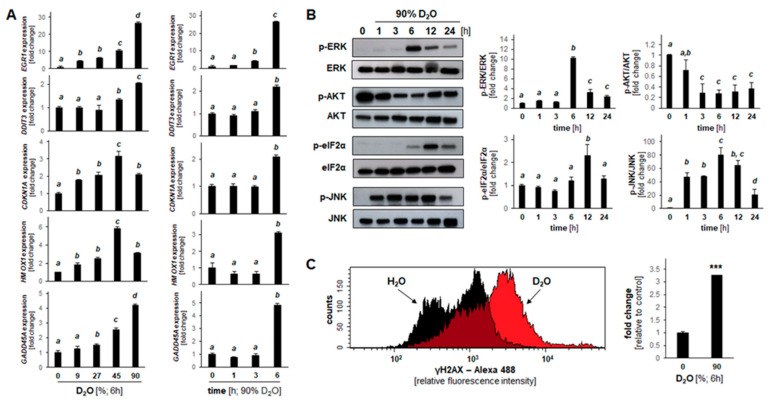
D_2_O-induced stress response gene expression and rapid onset of modulated phospho-protein signaling in A375 melanoma cells. (**A**) RT-qPCR assessment of gene expression; left row, dose response (≤90 % D_2_O); right row, time course (≤6 h). (**B**) Stress response protein phosphorylation in response to acute D_2_O exposure as profiled by immunoblot analysis: time course (90% D_2_O; ≤24 h). Bar graphs summarize quantitative analysis by densitometry (mean ± SD). (**C**) For γH2AX detection, flow cytometry was performed; bar graphs summarize quantitative analysis (mean ± SD; *n* = 3; p *** < 0.001).

Selected expression changes observable in both cell lines were also confirmed at the single RT-qPCR level (Figure 4A). Remarkably, no heat shock response related genes interrogated on the array (*HSPB1*, *HSPA6*, *HSPA1A*, *HSF1*, *HSPA4*, *HSPH1*, *HSPCA*, *HSPCB*, *HSPA5*, *HSPA2*, *DNAJA1*) displayed significant expression changes upon D_2_O exposure with the exception of *HSPA1L* and *HSPA8* that were downregulated in both A375 and PANC-1 cells. Taken together, these changes are consistent with the early induction of genotoxic, proteotoxic, oxidative, and inflammatory (*GADD45A*, *DDIT3*, *EGR1*, *HMOX1*, *SOD2*, *CCL4*, *CSF2*, *EGR1*, *NFKBIA*) stress responses resulting from D_2_O exposure [35,36,37,38,39]. 

### 2.4. D_2_O Modulates Stress Response Gene Expression and Protein Phosphorylation in A375 Melanoma Cells

After analysis of D_2_O-induced cellular responses and gene expression changes focusing on melanoma and PDAC cell lines (Figure 1, Figure 2 and Figure 3), we then focused exclusively on A375 cells for a more detailed examination of time-course and dose-response relationship substantiating a pronounced stress response inducible at the transcriptional level within hours (≤6 h) observable at low concentrations of D_2_O (≤27%) (Figure 4A). For example, upregulated expression of *EGR1*, *CDKN1A*, and *HMOX1* was detectable at D_2_O concentrations as low as 9% (6 h) (Figure 4A, left panels). Time-course analysis revealed that upregulated gene expression was detectable within 6 h exposure time except for *EGR1* observable already at 3 h (Figure 4A, right panels). Next, in order to substantiate D_2_O cellular responsiveness, immunodetection of D_2_O-induced phospho-protein signaling was performed (Figure 4B and Figure S3). To this end, A375 cells were D_2_O-exposed (90%; ≤24 h), followed by immunodetection of protein phosphorylation, focusing on a number of crucial components involving (i) AKT-, (ii) MAPK-, (iii) ER- (endoplasmic reticulum), and (iv) H2AX-related stress response pathways. Indeed, constitutive phosphorylation of AKT was rapidly suppressed; in contrast, ERK1/2- and JNK-MAPKs were found to undergo activational phosphorylation by D_2_O exposure. In contrast, p38-MAPK did not undergo significant phosphorylation changes (data not shown). In addition, it was observed that D_2_O exposure causes pronounced induction of ER-stress response signaling by phosphorylation of eIF2α (detectable within 6 h D_2_O exposure), an observation consistent with rapid induction of D_2_O-induced blockade of protein translation (Figure 4B and Figure S3).

In addition to alteration of AKT-, MAPK-, and eIF2α- phosphorylation status, γH2AX (i.e., phosphorylated (S139) H2AX generated by kinases (including ATM and ATR) in response to acute genotoxic stress) was detectable in response to short term D_2_O exposure (90%, 6 h) as detected by flow cytometry (Figure 4C). Taken together, these data indicate that acute D_2_O exposure performed at a wide dose range (≤90%) induces rapid and pronounced modulation of AKT-, MAPK-, ER-, and genotoxic stress response signaling detectable in A375 melanoma cells at the mRNA and phospho-protein levels. 

### 2.5. Systemic Administration of D_2_O Impairs Metastasis and Tumor Growth in SCID Mouse Models of Human Malignant Melanoma

**Figure 5:**
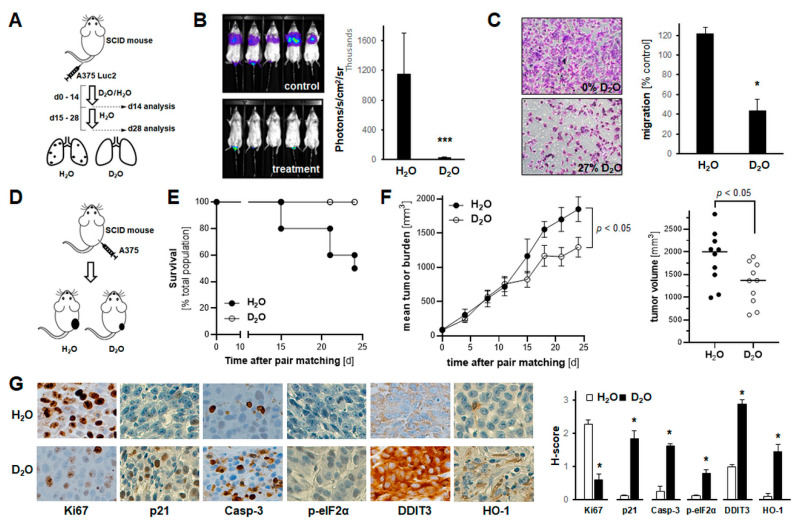
Systemic administration of D_2_O attenuates A375 melanoma cell invasiveness in vitro, while impairing metastasis and tumor growth in SCID mouse models of human malignant melanoma. (**A**,**B**) A375-Luc2 melanoma cells were tail vein injected (*n* = 8 per group) followed by bioluminescent image analysis of lung metastasis (14 and 28 days later). Starting at time of cell injection until day 14, mice received H_2_O-based or D_2_O-supplemented (30% *v*/*v* in H_2_O) drinking water, followed by 14 days H_2_O in both groups. A, injection scheme; B, bioluminescent imaging (day 28) with bar graph depicting numeric image analysis of bioluminescent signal (*p* *** < 0.001). (For bioluminescent imaging on day 14, see Figure S4). (**C**) Invasion through Matrigel-coated Boyden chamber (H_2_O-based versus D_2_O-supplemented (27%) medium). Bar graphs with representative images (10× magnification) after crystal violet staining of inserts (*n* = 3; *p* * < 0.05). (**D**–**G**) A375 melanoma cells were injected subcutaneously (*n* = 10 per group); after pair-matching, tumor growth was monitored over a 24-day period; starting at time of pair matching (day 0) until end of experiment (day 24), mice received H_2_O-based or D_2_O-supplemented (30% *v*/*v* in H_2_O) drinking water. (**D**) Experimental scheme. (**E**) Kaplan–Meier analysis of mouse survival as a function of treatment groups; numbers indicate survivors per group. (**F**) Tumor burden in treatment groups as a function of time; graph (right panel), individual tumor size at termination. (**G**) At the end of the experiment, tumors were processed for IHC (left panels; 20× magnification); bar graph: tissue H-scores per antigen (right panel; *n* = 3; *p* * < 0.05).

A bioluminescent in vivo model of melanoma lung metastasis as a function of systemic D_2_O administration (30% in drinking water; Figure 5A,B) was implemented following a previous drinking water regimen [5,9,10]. After tail vein injection, lung tumor burden imposed by A375-Luc2 melanoma cells was assessed using non-invasive bioluminescence imaging (day 14, Figure S4; day 28, Figure 5B). Lungs from animals receiving D_2_O-based drinking water displayed a diminished degree of bioluminescent signal indicative of attenuated lung metastasis (Figure 5B). Likewise, assessment of invasion through Matrigel-coated Boyden chambers (H_2_O versus D_2_O (27%)) indicated that D_2_O-treatment antagonizes A375 melanoma invasiveness in vitro (Figure 5C).

Next, it was examined if systemic administration of D_2_O impairs tumor growth in an SCID mouse xenograft model of human malignant melanoma (Figure 5D–G and Figure S5). To this end, A375 melanoma cells were injected subcutaneously, and animals received H_2_O-based or D_2_O-supplemented (30% *v*/*v* in H_2_O) drinking water starting at the time of pair matching and continued until the end of the experiment (Figure 5D). Tumor growth was then monitored over a 24-day period (Figure 5E–F). Kaplan–Meier analysis of mouse survival as a function of treatment groups revealed a dramatic extension of life span as a function of D_2_O supplementation (% survival per group: 50% (5 out of ten mice; H_2_O) versus 100% D_2_O)) (Figure 5E). Tumor burden was reduced in response to D_2_O supplementation displaying an approximately 30% average reduction versus the H_2_O group (Figure 5F). At the same time, no adverse effects of D_2_O supplementation were observable as evident from necropsy and liver panel data (not shown).

Moreover, immunohistochemical analysis of tumor tissue performed at the end of the experiment further substantiated D_2_O-induced anti-proliferative (Ki67, p21), pro-apoptogenic (cleaved caspase 3), and stress response (HO-1, DDIT3, p-eIF2α) modulatory activity detectable in A375 melanoma tumor-bearing SCID mice (Figure 5G and Figure S5). Taken together, these data support a significant chemotherapeutic role of D_2_O supplementation targeting melanoma tumor growth and metastasis observable in vitro and in vivo.

## 3. Discussion

Extensive prior investigations have explored biomolecular properties, toxicological relevance, and pharmacological potential of D_2_O, attributed mostly to isotope- (^2^H- versus ^1^H-) related changes in solvent effects, alteration of intramolecular hydrogen bond strength, and modulation of a limited number of specific molecular targets [10]. Numerous studies have explored potential chemotherapeutic effects of systemic D_2_O administration in murine models, but little information is available on D_2_O-responsive molecular pathways and efficacy relevant to metastasis and tumor growth [2,3,5,6,7,9].

Here, for the first time, we have profiled and compared the D_2_O-induced early stress response gene expression in A375 melanoma and PANC-1 pancreatic ductal adenocarcinoma cells (Figure 3), complemented by analysis of phosphoprotein stress signaling in A375 cells (Figure 4). We also demonstrate that systemic administration through drinking water supplementation suppresses lung tumor burden in a murine bioluminescent model of human malignant melanoma metastasis (Figure 5A,B); likewise, we report for the first time that D_2_O administration inhibits tumor growth while increasing median survival in a murine A375 xenograft model of human melanoma (Figure 5D–F). 

D_2_O-induction of apoptosis has been documented before in a variety of cultured cancer cell lines, involving modulation of apoptotic executioners (such as BAX and BCL2) and markers (including PARP-1 cleavage) through unknown upstream mechanisms responsive to D_2_O exposure [29,30,32]. Importantly, apoptogenicity of D_2_O targeting A375 melanoma cells and PANC-1 pancreatic ductal adenocarcinoma cells has been previously observed [9,40]. In our own investigations, using a panel of cultured malignant melanoma and pancreatic ductal adenocarcinoma cells, an apoptotic mode of cell death induced by D_2_O exposure was substantiated by annexin V-PI staining, pro-caspase 3 and PARP-1 cleavage, and pan-caspase inhibitor (zVAD-fmk) rescue, observed in both A375 melanoma and PANC-1 pancreatic ductal adenocarcinoma cells (Figure 1 and Figure 2). Also, using a genetic model of BRAF^V600E^ kinase inhibitor resistance employing the A375^NRAS^ isogenic melanoma cell line, our data indicate that D_2_O sustains activity irrespective of BRAF mutational status (an observation suggesting therapeutic efficacy targeting kinase-resistant melanoma cells using D_2_O (Figure 1A,E)).

In an attempt to provide avenues for future investigations that would identify specific molecular targets and mechanisms underlying the potential chemotherapeutic effects of D_2_O we aimed at identifying upstream stress response pathways that might be sensitive to D_2_O exposure. To this end, we employed differential expression array analysis of D_2_O-exposed cells performed at an early time point (6 h), characterized by complete maintenance of cell viability (before occurrence of D_2_O-induced cell death detectable upon prolonged exposure (24 h)) (Figure 1, Figure 2 and Figure 3). Remarkably, early modulation of a signature stress response gene expression pattern was observable in both A375 melanoma and PANC-1 pancreatic ductal adenocarcinoma cells (Figure 3). Likewise, in A375 melanoma cells, immunoblot analysis revealed rapid onset of D_2_O-induced stress response phospho-protein modulation (involving downregulation of p-AKT and upregulation of p-ERK, p-JNK, p-eIF2α and p-H2AX) (Figure 4). It is remarkable that our phosphoprotein analysis indicated pronounced activation of ERK and JNK signaling, contrasted by early downregulation of p-AKT, changes known to orchestrate melanoma cell survival and resistance to apoptosis (Figure 4B). Specifically, p-JNK has been shown to play an essential role in drug-induced melanoma cell apoptosis, and p-AKT has been identified as a major driver of melanomagenesis [41,42]. Thus, D_2_O modulation of AKT phosphorylation (as substantiated here for the first time) is reminiscent of earlier reports suggesting that AKT represents a molecular target exquisitely sensitive to modulation of hydrogen bond networks [24,25]. Moreover, our comparative array analysis interrogating D_2_O-induced expression of heat shock response genes in A375 and PANC-1 cells detected the downregulated expression of two heat shock protein encoding genes (*HSPA8* and *HSPA1L*) that occurred in the striking absence of any other heat shock related gene expression changes, combined with phosphorylational activation of the ER-stress sensor eIF2α and its downstream transcriptional target *DDIT3* (encoding CHOP) (Figure 3 and Figure 4).

Deuterium isotope effects on non-covalent biomolecular interactions have been traditionally attributed to hydrogen bond alterations and solvent effects, relevant to function of nucleic acids and proteins [13,14,17,43]. Indeed, hydrogen bonds are subject to isotope effects, and in water D-bonds are stronger than H-bonds (by approximately 0.1 to 0.2 kcal mol^-1^), attributed to the higher mass of deuterium that lowers the zero-point vibrational energy. Likewise, intrapeptide D-bonds are presumably stronger than H-bonds, increasing protein rigidity due to conformational stabilization; in addition, it has long been recognized that solvent effects (due to a greater enthalpic D_2_O–D_2_O affinity) impact protein and nucleic acid stability causing conformational alterations and aggregation changes due to increased hydrophobic effects [13,14,15,17,43]. For example, consistent with physicochemical biomolecular effects of D_2_O (i.e., alteration of hydrogen bond strength causing protein conformational rigidity attributed to strengthening of hydrogen bonds), we observed the significant absence of heat-shock-related gene expression changes (Figure 3C,D), an observation supported by earlier reports documenting the D_2_O-based attenuation of heat shock response induction and heat shock protein expression attributed to D_2_O-suppression of protein unfolding [44]. Likewise, D_2_O-induced anti-proliferative effects observed by us in A375 malignant melanoma cells in vitro and in vivo (including M-phase depletion (Figure 1D), *CDKN1A* upregulation (Figure 4A and Figure 5G), and Ki67 downregulation (Figure 5G)) might be attributed to mitotic interference and potential nucleic acid-directed alterations of hydrogen bond-based interactions impairing nucleic acid replication and transcription, a finding consistent with earlier reports [10,28,32,40,45,46]. Likewise, early γH2AX signaling might be indicative of genotoxic stress induced by D_2_O-exposure, further substantiated by pronounced *GADD45* upregulation (Figure 4A,C). Interestingly, *GADD45A* expression was affected in opposing ways when comparing A375 and PANC-1 cells, a phenomenon to be explored mechanistically in future experiments.

Importantly, in spite of our identification of D_2_O-responsive early response gene expression changes and phosphoprotein signaling, the molecular mechanism underlying D_2_O-induced cellular responses, shared between PANC-1 and A375, remains to be explored in much detail. In the context of cancer-directed molecular mechanisms, it will be of particular interest to test a causative involvement of previously identified D_2_O-targets such as aquaporin 11 (AQP11; identified in activated hepatic stellate cells) and L-type calcium channels (identified in rat thoracic aorta smooth muscle cells) relevant to non-oncological physiological D_2_O effects such as modulation of blood pressure [10,16,18,19,20,21,22,23,26,47].

Molecular stress response dysregulation has been identified as a hallmark of tumorigenesis representing a valid target for therapeutic intervention, but the role of D_2_O-induced stress response modulation targeting cancer cells has remained largely unexplored [10,33,34]. D_2_O has been administered to humans before reaching 23% tissue saturation without induction of toxicity, and, in murine models, oral administration of D_2_O (at 20% and higher) was associated with significant inhibition of tumor growth [2,3,5,6,7,8,9,10,11,12]. Therefore, in order to explore the translational potential of D_2_O-based chemotherapeutic intervention in human patients, essential data establishing a safe therapeutic window will be needed, also in the context of potential D_2_O combinatorial chemosensitization regimens with established chemotherapeutics as demonstrated before in cell culture and relevant mammalian cancer models [6,9,40]. Based on our data documenting efficacy of D_2_O-based interventions targeting malignant melanoma (in vitro and in vivo) and also PDAC (in vitro) together with upstream gene expression changes, our future research will aim at elucidating: (i) target identity, (ii) therapeutic window, (iii) dose regimen, and (iv) combinatorial use with current chemotherapeutics potentially benefitting human cancer patients in the near future.

## 4. Materials and Methods 

### 4.1. Chemicals

All chemicals and reagents were purchased from Sigma Aldrich Chemical Co (St. Louis, MO, USA), unless specified otherwise. 

### 4.2. Cell Culture

The following cell lines were purchased from ATCC (Manassas, VA, USA): human malignant melanoma cells (A375 (CRL-1619), A375-Luc2 (containing BRAF V600E mutation; CRL-1619-LUC2), NRAS-mutant-A375-Luc2 (isogenic variant containing both BRAF V600E and NRAS Q61K mutations; CRL-1619IG-2-LUC2), and G361 (CRL-1424)), human pancreatic ductal adenocarcinoma cells (PANC-1-Luc2 (CRL-1469-LUC2), MIA PaCa-2 (CRL-1420), and Capan-2 (HTB-80)), and normal skin fibroblasts (Hs27 (CRL-1634)), cultured under standard conditions as specified by the manufacturer [48,49,50]. LOX-IMVI melanoma cells (SCC201) were purchased from Millipore Sigma. For D_2_O exposure in culture media, respective powder medium (RPMI/Roswell Park Memorial Institute, DMEM/Dulbecco’s Modified Eagle Medium, McCoy’s 5a; 4.5–90.0% (*v*/*v*)) was reconstituted using either H_2_O (Milli-Q^TM^ Ultrapure, Millipore, Burlington, MA, USA) or D_2_O (analytical grade, #151882, Sigma Aldrich), with 10% FBS. 

### 4.3. RNA Extraction and Single Reverse Transcrition Quantitative Polymerase Chain Reaction (RT-qPCR)

Cellular total RNA was isolated individually using Qiagen RNeasy Mini Kit (Qiagen Sciences, Gaithersburg, MD, USA) and RT-qPCT was performed according to the manufacturer’s protocol as published before [51,52]. The following primer probes were used: human EGR1 (Hs_00152928_m1, FAM), DDIT3 (Hs_00358796_g1, FAM), CDKN1A (Hs_00355782_m1; FAM), HMOX1 (Hs_00157965_m1; FAM), GADD45A (Hs_00169255_m1, FAM), and RPS18 (housekeeping gene; Hs_01375212_g1; VIC); 20X primer/probes were obtained from Thermo Fisher Scientific, Waltham, MA, USA. Expression levels of EGR1, DDIT3, CDKN1A, HMOX1, and GADD45A were normalized to the RPS18 control (ΔCt = Ct (gene of interest) − Ct (housekeeping gene)).

### 4.4. Immunoblot Detection

Cellular protein extraction, 4–15% gradient SDS-PAGE gel electrophoresis (Bio-Rad laboratories, Irvine, CA, USA), transfer to PVDF membrane, and immunoblot development were performed as published recently [37]. The following rabbit anti-human antibodies were used (obtained from Cell Signaling, Danvers, MA): cleaved PARP (5625), p-ERK (4370), ERK (4695), p-AKT (4060), AKT (4691), p-eIF2α (3398), eIF2α (5324), p-SAPK/JNK (4668), and SAPK/JNK (9252). Equal protein loading was examined by β-actin detection using a mouse monoclonal antibody (Sigma Aldrich); secondary antibodies: HRP-conjugated goat anti-rabbit or goat anti-mouse (Jackson ImmunoResearch Laboratories, West Grove, PA, USA). Densitometric image analysis was performed using Image Studio^TM^ Lite quantification software (LI-COR Biosciences, Lincoln, NE, USA).

### 4.5. Flow Cytometric Analysis of Cell Viability

After D_2_O (up to 90%; up to 6 days) treatment, viability of melanoma and pancreatic cancer cells was assessed by annexinV-FITC/propidium iodide (PI) dual staining followed by flow cytometric analysis as published before using an apoptosis detection kit according to the manufacturer’s specifications (APOAF, Sigma Aldrich, St. Louis, MO, USA) [48,49].

### 4.6. Cell Proliferation Assay

Cell numbers at the time of D_2_O addition (day 0) and 72 h later (day 3) were determined using a Z2 Analyzer (Beckman Coulter, Fullerton, CA, USA), and proliferation was compared with cells that received mock treatment following our published procedure [50,53].

### 4.7. M-Phase Quantification by Phospho-Histone H3 (Ser10) Flow Cytometry

Using a rabbit derived Alexa-488 conjugated antibody (Cell Signaling), cells in M-phase were detected by bivariate flow cytometric determination of cellular DNA content (PI-staining) and histone H3 phosphorylated at Ser 10 (p-H3(Ser10)); p-H3(Ser10)-positive cells were expressed in percent of total gated cells as published before [49,50].

### 4.8. Caspase-3 Activation Assay

D_2_O-induced caspase-3 activation was examined by flow cytometric analysis using a cleaved/activated caspase-3 (asp 175) antibody (Alexa Fluor 488 conjugate, Cell Signaling) following our published procedure [50].

### 4.9. Cytometric Assessment of Histone H2AX Phosphorylation

D_2_O-induced accumulation of the nuclear phosphorylated histone variant H2AX (γH2AX) was examined using a phospho-histone H2AX (Ser139) Alexa Fluor 488 conjugated monoclonal antibody (Cell Signaling) followed by flow cytometric analysis according to our published procedure [50]. 

### 4.10. Comparative RT^2^ Profiler^TM^ PCR Gene Expression Array Analysis

The human Stress and Toxicity Pathway Finder RT^2^ Profiler^TM^ technology (Qiagen) assessing expression of 84 stress response regulatory genes (normalized to a group of five housekeeping genes (*ACTB*, *B2M*, *GAPDH*, *HPRT1*, and *RPLP0*)) was employed following our published procedures [49,53].

### 4.11. Transwell Invasion Assay

To evaluate the cellular invasion potential, a published standard procedure was followed [49,52,54]. Either normal growth medium (10% FBS; 0% D_2_O) or D_2_O supplemented medium (10% FBS; 27% D_2_O) was added to the bottom of each well and a total of 4 × 10^4^ cells resuspended in invasion buffer (0.5% FBS; 0.1% BSA) were seeded on top. The number of invading cells was quantified 24 h later by counting 10 random fields per filter.

### 4.12. Metastasis Model in SCID Mice

A375-Luc2 melanoma cells (1 × 10^6^ cells resuspended in 100 μL Hank’s balanced salt solution) were administered to SCID mice (obtained from the University of Arizona Cancer Center SCID mouse colony at the age of 9 weeks with an average weight of 20 g (*n* = 8 per group)) by intravenous (i.v.) tail vein injection [49]. Starting at time of cell injection (until day 14 post injection), mice received H_2_O-based or D_2_O-supplemented (30% *v*/*v* in H_2_O) drinking water, followed by H_2_O supplementation for another 14 days. Bioluminescent image analysis of lung metastasis occurred on days 14 and 28. This study was performed in accordance with the recommendations of the National Institutes of Health (University of Arizona Institutional Animal Care and Use Committee; mouse protocol number: IACUC 17–298).

### 4.13. Xenograft Model in SCID Mice

Using an SCID mouse colony established at the University of Arizona (originating from SCID C.B-17/IcrACC SCID obtained from Taconic (Germantown, NY, USA)), A375 cells (10 × 10^6^) were injected subcutaneously on day 0 (*n* = 10 per group). One group was receiving normal drinking water while the other group was receiving water supplemented with 27% D_2_O for the duration of the whole experiment [5,9,10]. Tumor growth curves were generated by monitoring average tumor volumes (mm^3^) until day 24 after cell injection followed by tumor collection [49,54]. All procedures were completed in accordance with the University of Arizona Institutional Animal Care and Use Committee protocol (IACUC 17–298). 

### 4.14. Immunohistochemistry

After tumor collection, tissue was processed for immunohistochemical analysis following our published procedures [49,52]. Antigen detection was performed using the following antibodies: Ki67 (ab15580; Abcam, Cambridge, MA, USA), p21 (2947; Cell Signaling, Danvers, MA, USA), cleaved Casp-3 (9661, Cell Signaling), phospho-eIF2α (3398; Cell Signaling), DDIT3 (179823; Abcam), and HO-1 (ab13248; Abcam). Slides were scored manually using a 20 × objective. Average histologic scores (H-score) were calculated as previously reported [55]. Based on the percentage of cells staining with 3+ (strong), 2+ (moderate), 1+ (weak), or O (absent) intensity, an H-score (range 0–3) was calculated by summing the percentages of cells staining at each intensity multiplied by the weighted intensity of staining: H-score = (% weakly stained cells × 1) + (% moderately stained cells × 2) + (% strongly stained cells × 3).

### 4.15. Statistical Analysis

Numerical data were analyzed as published recently [49,56]. Unless stated differently, data sets were analyzed employing analysis of variance (ANOVA) with Tukey’s post-hoc test using the Prism 8.4.3 software (Prism Software Corp., Irvine, CA, USA); in respective bar graphs (analyzing more than two groups), means without a common letter differ (*p* < 0.05). For bar graphs comparing two groups only, statistical significance was calculated employing the Student’s two-tailed t-test, utilizing Excel (Microsoft^TM^, Redmond, WA, USA). Experiments involved at least nine individual replicates per data point, except for gene expression array analysis performed with three independent biological replicates. Nonparametric data analysis of murine experimentation was performed using the Mann–Whitney test. The level of statistical significance was marked as follows: *p* * < 0.05; *p* ** < 0.01; *p* *** < 0.001.

## 5. Conclusions

Here, we have profiled the anti-proliferative and apoptogenic effects of deuterium oxide exposure together with stress response gene expression employing panels of cultured malignant melanoma and pancreatic ductal adenocarcinoma cells. Moreover, we have substantiated the efficacy of D_2_O-based pharmacological intervention in murine models of human melanoma tumor growth and metastasis. Based on these explorative investigations, future research will aim at elucidating specific molecular mechanisms and translational feasibility of therapeutic D_2_O administration, potentially benefitting human cancer patients in the nearer future.

## Data Availability

The data presented in this study are available on request from the corresponding author.

## Supplementary Materials

The following are available online at https://www.mdpi.com/2072-6694/13/4/605/s1, Figure S1: The uncropped blots and molecular weight markers of Figure 1G; Figure S2: The uncropped blots and molecular weight markers of Figure 2D; Figure S3A–D: The uncropped blots and molecular weight markers of Figure 4B (Figure S3A: ERK; Figure S3B: AKT; Figure S3C: eIF2α; Figure S3D: JNK); Figure S4: Bioluminescent imaging data acquired at the end of the two weeks D2O regimen complementing data in Figure 5B; Figure S5: Representative ‘low power’ IHC images of tumor tissue complementing data in Figure 5G.
