# Supplementary material for: Deuterium Oxide (D2O) Induces Early Stress Response Gene Expression and Impairs Growth and Metastasis of Experimental Malignant Melanoma
Source: Cancers (Basel). 2021 Feb 3;13(4):605. doi: 10.3390/cancers13040605 (PMC7913703; doi:10.3390/cancers13040605)

Figure S1. The uncropped immunoblots and molecular weight markers of Figure 1G.

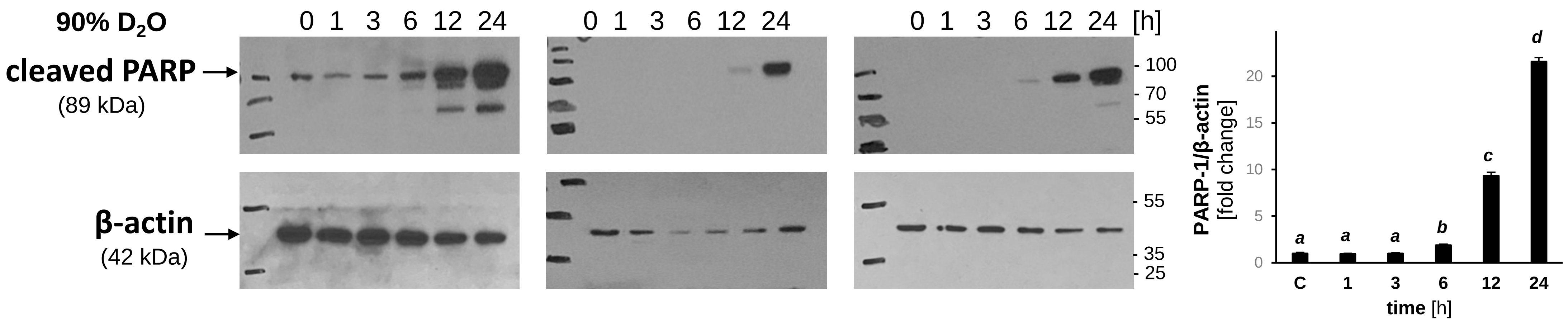

Figure S2. The uncropped immunoblots and molecular weight markers of Figure 2D

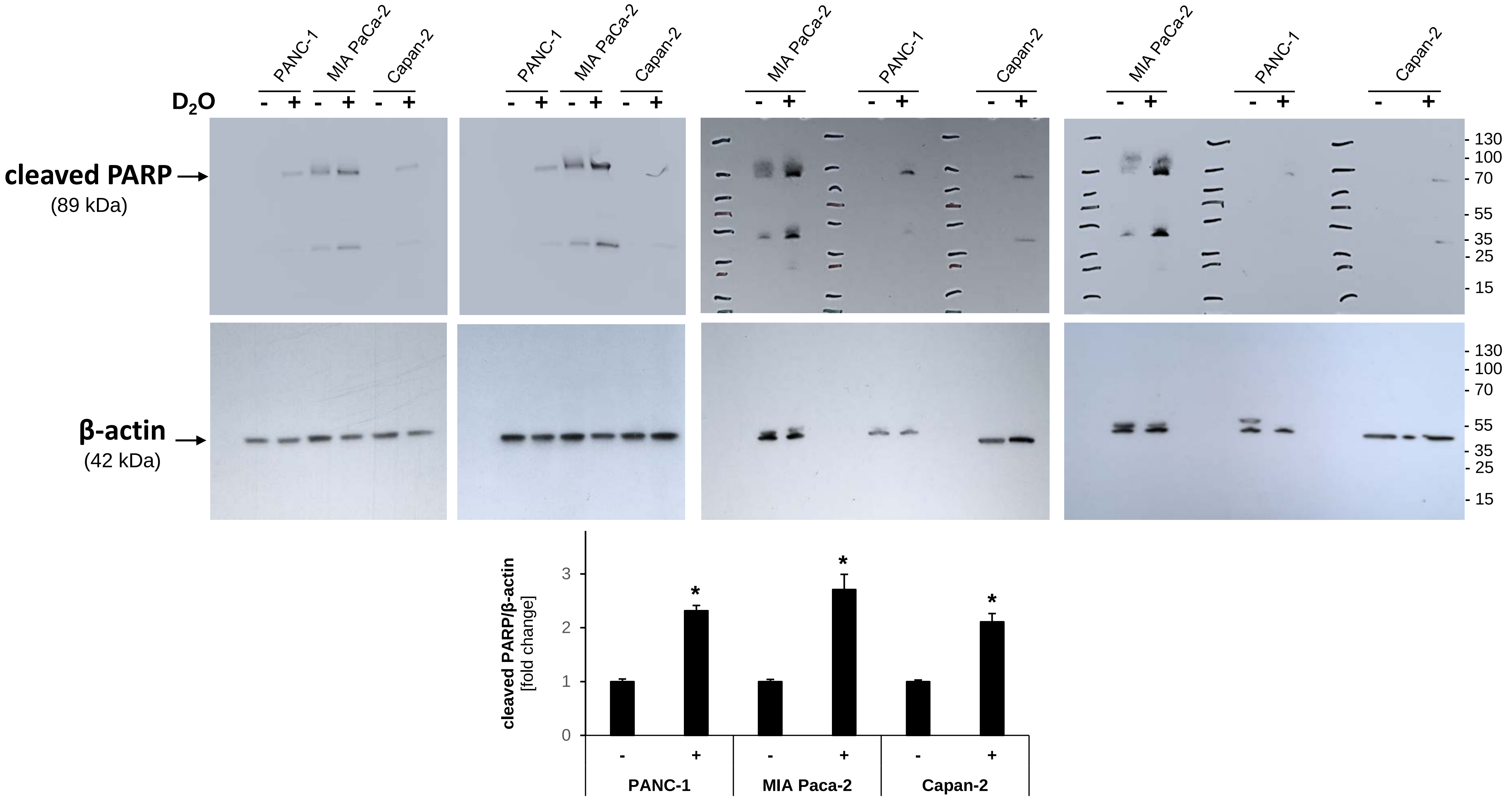

Figure S3A. The uncropped immunoblots and molecular weight markers of Figure 4B (antigen: ERK)

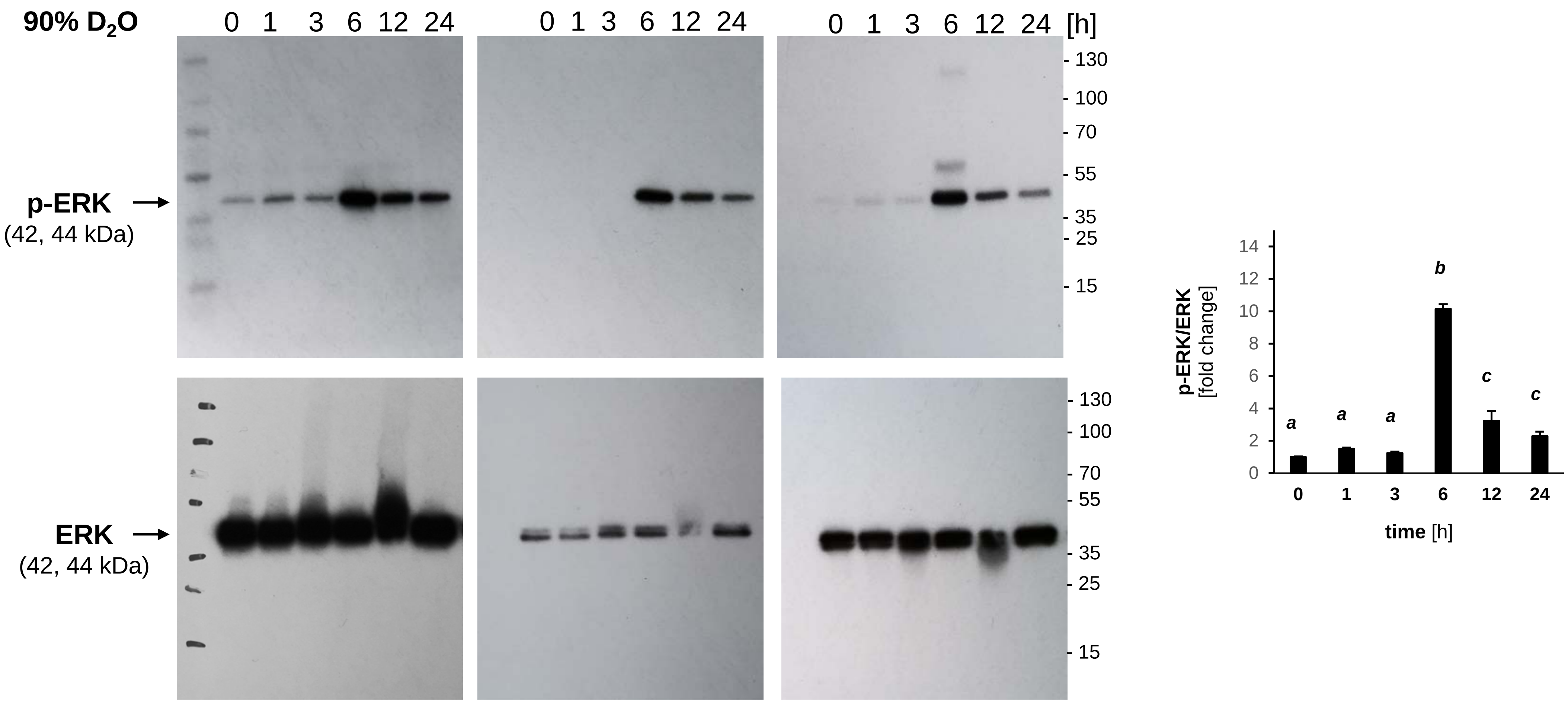

Figure S3B. The uncropped immunoblots and molecular weight markers of Figure 4B (antigen: AKT)

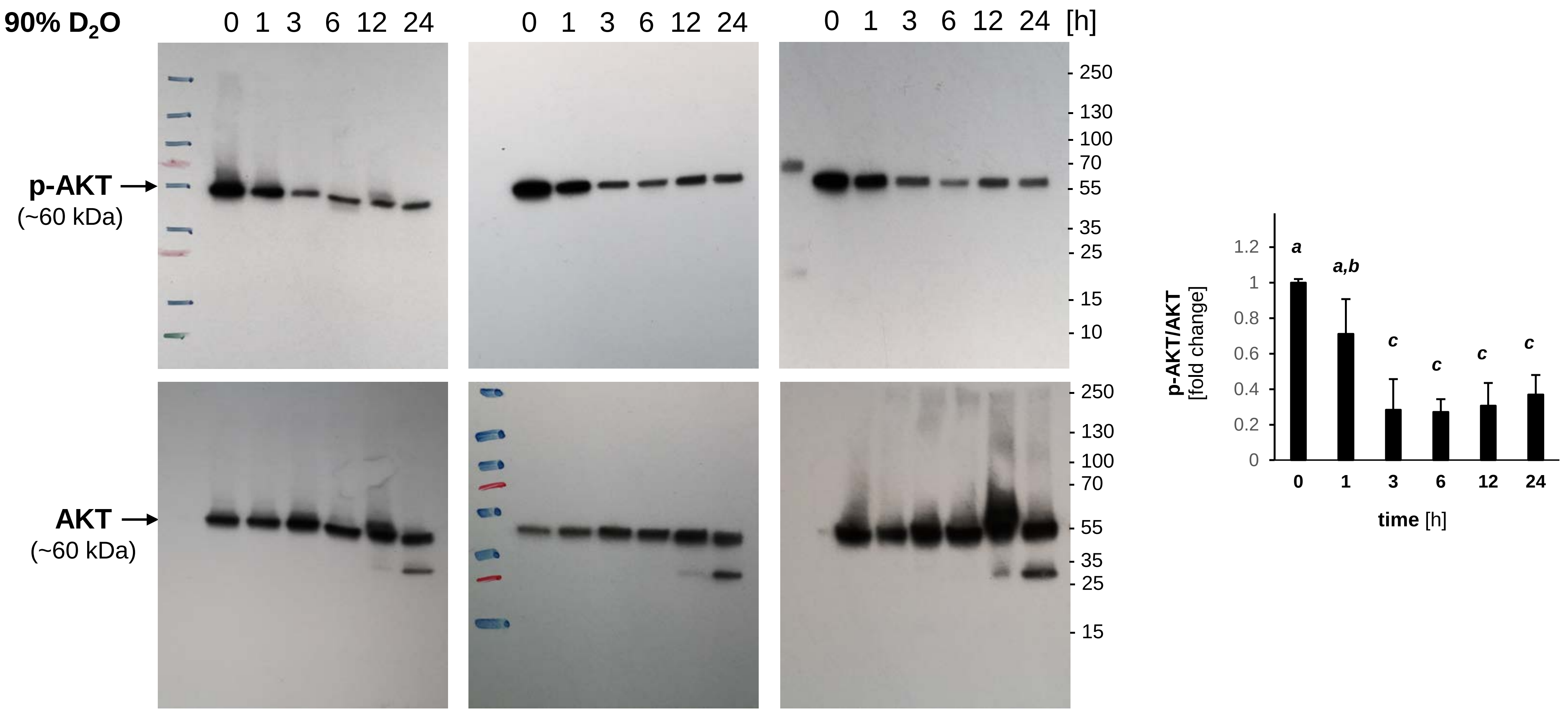

Figure S3C. The uncropped immunoblots and molecular weight markers of Figure 4B (antigen: eIF2α)

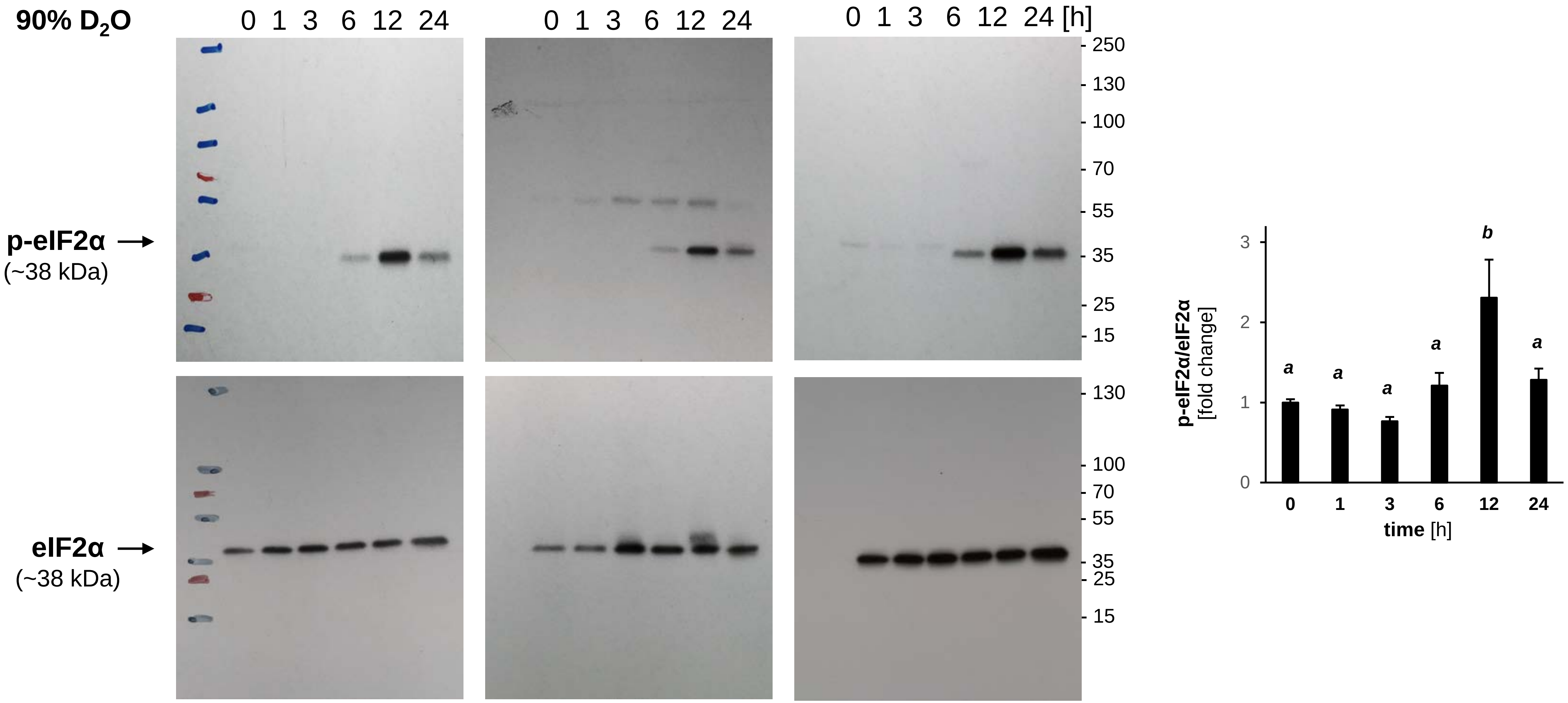

Figure S3D. The uncropped immunoblots and molecular weight markers of Figure 4B (antigen: JNK)

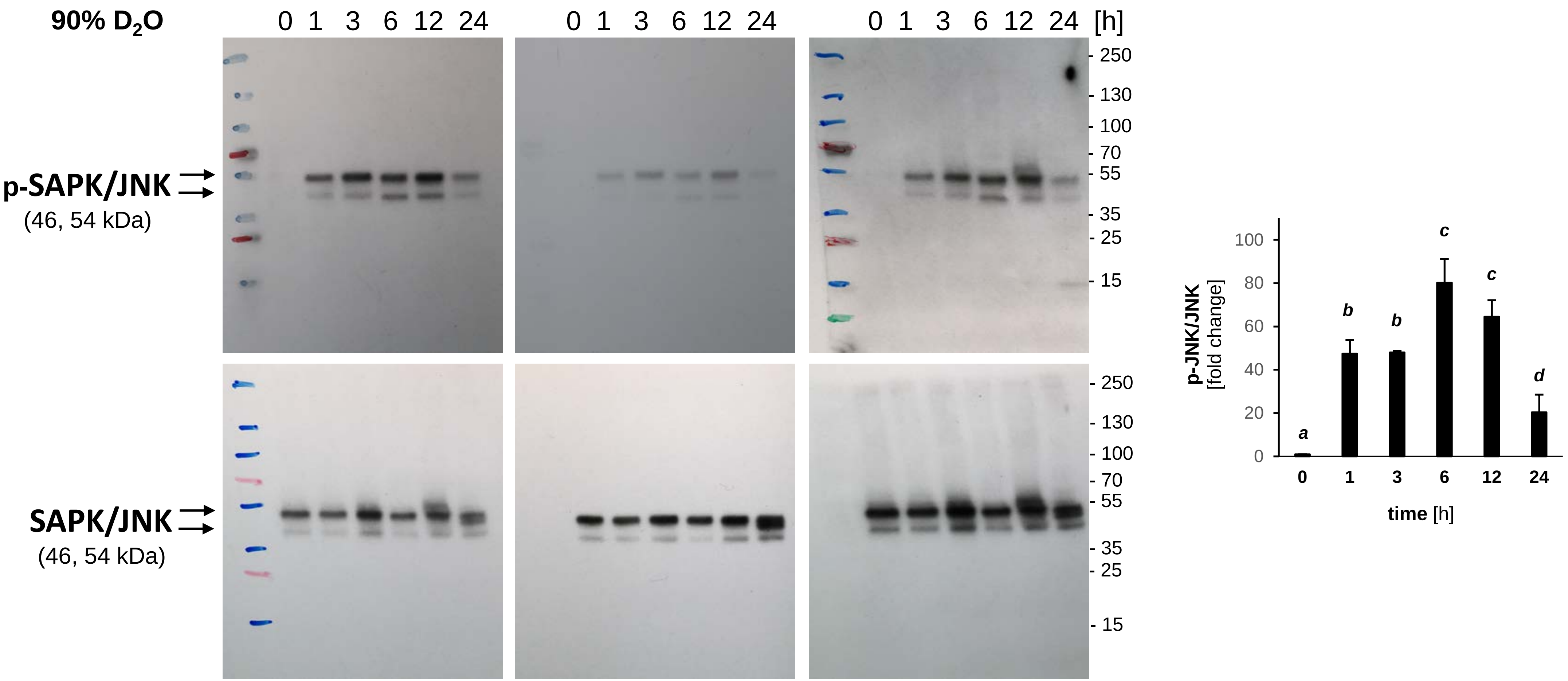

Figure S4. Systemic administration of D_2O impairs A375 melanoma cell metastasis in a SCID mouse model. Bioluminescent imaging data acquired at the end of the two weeks D_2O regimen. Luminescent cells were injected at day 0, mice were then supplemented with drinking water (D_2O vs. H_2O) until day 14, and imaging occurred on day 14; (see Fig. 5 for experimental details and day 28 data).

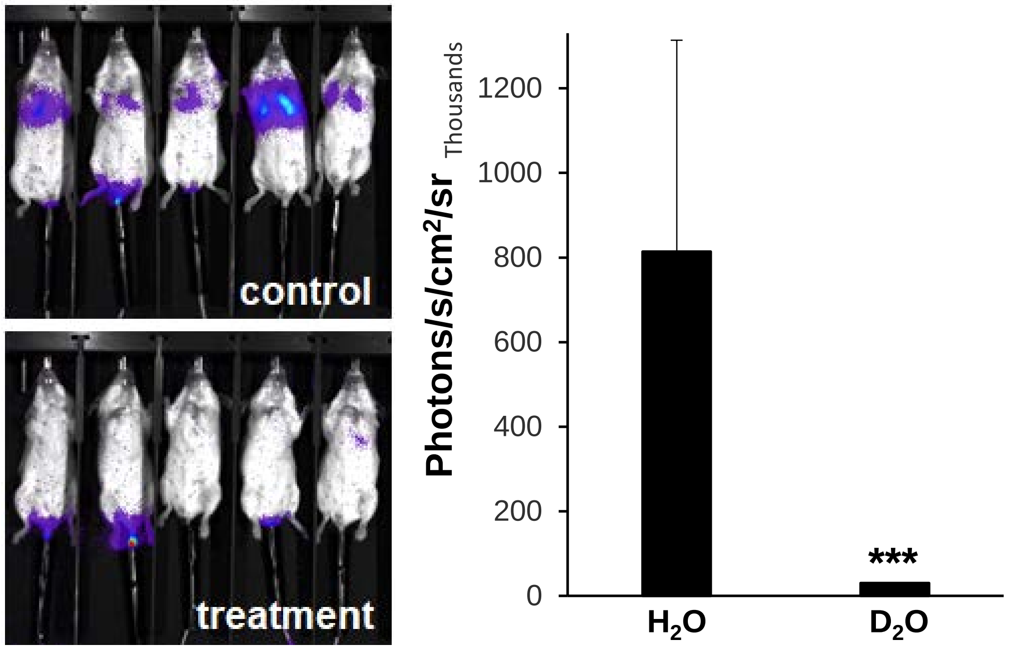

Figure S5. Representative 'low power' IHC images of tumor tissue stained for the following antigens: Ki67, DDIT3 and HO-1 (see legend of Fig. 5G for details on tumor specimens).

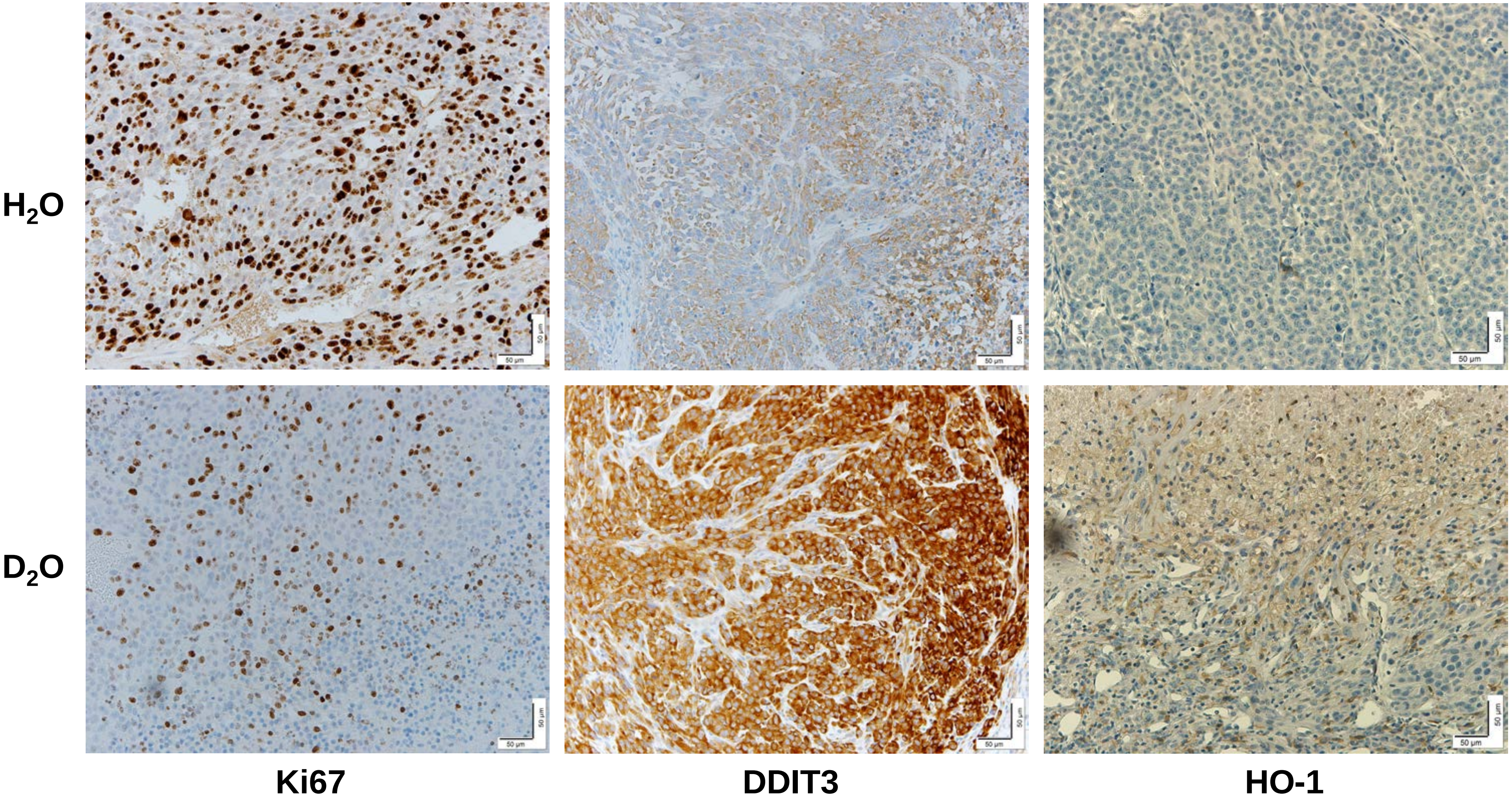

Supplement: Supplementary file 1 [file cancers-13-00605-s001.pdf]
